# The role of whole exome sequencing in the UBE3A point mutation of Angelman Syndrome: A case report

**DOI:** 10.1016/j.amsu.2021.103170

**Published:** 2021-12-08

**Authors:** Agung Triono, Kristy Iskandar, Andika Priamas Nugrahanto, Marissa Leviani Hadiyanto, Gunadi, Elisabeth Siti Herini

**Affiliations:** a Department of Child Health, Faculty of Medicine, Public Health and Nursing, Universitas Gadjah Mada, Dr. Sardjito Hospital, Yogyakarta, 55281, Indonesia; b Genetics Working Group, Faculty of Medicine, Public Health and Nursing, Universitas Gadjah Mada, Dr. Sardjito Hospital, Yogyakarta, 55281, Indonesia; c Pediatric Surgery Division, Department of Surgery, Faculty of Medicine, Public Health and Nursing, Universitas Gadjah Mada, Dr. Sardjito Hospital, Yogyakarta, 55281, Indonesia

**Keywords:** Angelman syndrome, Point mutation of UBE3A gene, Whole-exome sequencing, Limited resources, Case report

## Abstract

**Introduction:**

Angelman Syndrome (AS) is a rare disorder with a relatively well-defined phenotype caused by lack of expression of the maternally inherited ubiquitin-protein ligase E3A (*UBE3A*) gene in the brain. This article describes the role of genetic testing using whole-exome sequencing (WES) in detecting rare AS variants, a point mutation in the UBE3A gene.

**Case presentation:**

We describe a rarely reported clinical presentation of AS in a two year and ten months old girl with severe developmental delay, movement and balance disorder, frequent smiling, apparent happy demeanor, speech impairment, absence of seizure, lack of sleep, and abnormal food-related behavior. Physical examination showed microcephaly, with facial characteristics of AS, ataxia gait, and truncal hypotonia. The electroencephalogram showed medium amplitude rhythmic 2-3c/s. Brain Magnetic Resonance Imaging revealed microcephaly, corpus callosum dysgenesis, and heterotopia grey matter on the bilateral lateral ventricle. WES was conducted to search pathogenic variants and showed a heterozygous mutation in exon 9 of the U*BE3A* gene, c.1513C > T (p.Arg505Ter).

**Conclusion:**

Angelman syndrome is a neurodevelopmental disorder that has several underlying genetic etiologies. WES could detect a rare variant of Angelman syndrome, identified as the point mutation of the *UBE3A* gene, which cannot be seen with other modalities.

## Highlights

• Angelman syndrome is a rare neurodevelopmental syndrome characterized by developmental delay, movement or balance disorder, tendency to laugh easily, and speech impairment.
• We reported a rare case of Angelman Syndrome, a point mutation in the UBE3A gene using the Whole-exome sequencing (WES) method that cannot be detected by methylation test.

## 1 Introduction

Angelman Syndrome (AS) is an uncommon neurodevelopmental syndrome marked by characteristic facial features, significant developmental delays with motor dysfunction, speech difficulty, a high incidence of epilepsy, and sleep and eating difficulties [1,2]. AS is associated with mutations in the *UBE3A* gene, which is inherited from the mother. There is four known genetic mechanisms of AS: (1) deletion in chromosome 15q11-q13 (70% of cases), (2) uniparental paternal disomy (UPD; 2% of cases), (3) imprinting defect (3% of cases), and (4) point mutation or maternally inherited *UBE3A* mutations (10% of cases) [[2], [3], [4]]. This syndrome has a long-term, major negative impact on the quality of life of children and their families. The prevalence ranges from 1 in 10,000–24,000 births [1,3,4]. The severity of the phenotype depends on the type of mutation that occurs, with a point mutation of the *UBE3A* gene will have a milder phenotype than those with deletion, UPD, or imprinting defects [4,5].

Whole-Genome Sequencing (WES) is recently being used because it can detect pathogenic small nucleotide variants in the *UBE3A* gene, uniparental isodisomy, and certain large deletions [6] In this paper, we reported a two year and ten months old girl with Angelman syndrome due to heterozygous mutation in exon 9 of the UBE3A gene, c.1513C > T (p.Arg505Ter), which was diagnosed using WES. We also described the importance of WES for diagnosing AS, particularly in a limited resource setting.

## 2 Case presentation

### 2.1 History

A 2 year and ten months old girl was referred to our hospital due to developmental delays and speech impairment. The mother realized that her child had developmental delays at 18 months and was referred to our hospital seven months later. She was the first child of non-relatively healthy parents with normal prenatal and birth history. She has a younger sister with normal development. The patient was born at 37 weeks of gestation, with a birth weight of 3,500 g, length of 50 cm, and head circumference of 33 cm. She showed mild developmental delays in the first year of life: head control at three months, sitting at seven months, and crawling at nine months. At almost three years old, she could only walk 2–3 steps and tended to fall backed due to truncal hypotonia.

She tends to laughed easily, had a happy demeanor, hyperactive behavior, and attention deficits. The patient often experiences absence seizures for about <5 seconds and 1–2 times per day since 2.5 months old. The patient had a sucking disorder since the age of 3 months, drooling excessively, and a habit of putting inedible things into her mouth. She had a good appetite and did not show any feeding problems. She also acted very interested in water and had sleep deprivation.

### 2.2 Physical examination

**Fig. 1:**
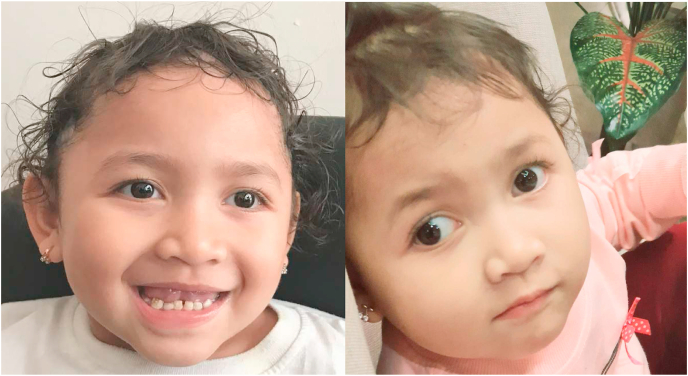
Clinical features of the 3 years old patient with AS showed wide smile, wide-spaced teeth, strabismus, with hypopigmented skin and hair.

The child had a normal nutritional status, microcephaly, wide mouth, protruding tongue, wide-spaced teeth, strabismus, hypopigmented skin, and hair according to a physical examination. Typical facial features in Angelman syndrome were shown in Fig. 1. Characteristically, she kept his hands uplifted, flexed arm position, especially during walking with valgus-positioned ankles. During the examination, she was very happy and had a social disposition. She also liked to observe and play with objects around her with constant interest. Neurological examination revealed significant intellectual impairment, ataxia, unsteady gait, receptive and non-verbal communication abilities superior to her verbal communication abilities, hypertonus of the limbs with hyperactive deep tendon reflexes, and truncal hypotonia.

### 2.3 Additional and radiology examination

**Fig. 2:**
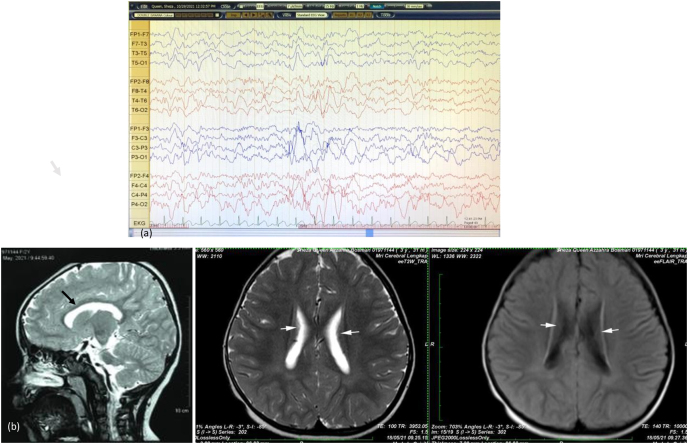
(a) EEG recording showing abnormal pattern during sleep with medium amplitude rhythmic 2-3c/s. (b) Sagittal and axial brain MRI showed microcephaly, corpus callosum dysgenesis, and heterotopia grey matter on the bilateral lateral ventricle.

The electroencephalogram (EEG) with the characteristic pattern for AS is showed in Fig. 2a. The brain's Magnetic Resonance Image (MRI) is shown in Fig. 2b with microcephaly, corpus callosum dysgenesis, and heterotopia grey matter on the bilateral lateral ventricle. The brainstem evoked response audiometry test was normal.

### 2.4 Genetic analysis

Blood sample was collected from the patient to obtain genomic DNA. xGen Exome Research Panel v2 caught all exon regions of all human genes (22,000) (Integrated DNA Technologies, Coralville, Iowa, USA). Novaseq 6000 was used to sequence the capture areas of the genome (Illumina, San Diego, CA, USA). The raw exome sequencing data, including alignment to the GRCh37/hg19 human reference genome, variant calling, and annotation, was performed using open-source bioinformatics tools and custom software. To prioritize variations based on ACMG guidelines, in-house created software, EVIDENCE, was used to automatically interpret variants and each patient's phenotype. This technique consists of three primary steps: variation filtering, categorization, and rating of patient phenotypic similarity. Finally, the potential variations and diseases that they are connected to will be manually analyzed [7,8].

WES showed a pathogenic variant identified as a heterozygous mutation in exon 9 of the *UBE3A* gene, c.1513C > T (p.Arg505Ter). Parents were counseled, and child was offered multipronged therapy. Since having absence seizures, the patient was given valproic acid (15 mg/kgBW/day). The patient had received walking physiotherapy and speech therapy for three months but had not shown significant improvement.

## 3 Discussion

Angelman syndrome was initially identified in 1965 and is often referred to as the ‘happy puppet’ syndrome [9]. To our knowledge, this is the first documented case of AS in Indonesia. This may happen because genetic testing tools are not routinely conducted in our country. There is a lack of funding support for genetic testing, shortage of competent health care staff to do the testing, and the national health insurance has not made genetic testing a standard diagnostic test.

The consensus criteria described four typical clinical features consistent (100%) in AS patients, also present in our patient [9]. The patient had developmental delays, movement or balance disorder, behavioral uniqueness with any combination of frequent laughter/smiling, and speech impairment. Clinical characteristics found in >80% of AS patients were also found in our patient: seizures, abnormal EEG, and microcephaly.

Currently, the patient has no difficulty eating and has a good appetite. This is in accordance with another case report of AS that showed there was no difficulty in eating syndrome [10,11] However, at three months old, our patient had difficulty sucking. Feeding problems usually occur in the first six months of life, such as uncoordinated sucking, poor breast attachment, and tongue thrusting. In later infancy, they usually experience gastroesophageal reflux, constipation, esophagitis, excessive swallowing, and cycling vomiting [9,12].

Seizures are usually present before the age of three years, while in our case, the seizure type manifested as an absence seizure, which her parents had difficulty recognizing at first [13]. In a study conducted in the United States, absence seizures were equally observed in almost patients with AS, while generalized motor seizures were more frequently observed in subjects with deletions [14]. The presence of a cluster of genes coding for three subunits of the GABA receptor complex in the usually deleted region, 15q11-q13, has suggested that GABA neurotransmission has a role in AS [15].

In most cases, EEG recordings in AS are very abnormal, and even if there are no clinical seizures, the EEG abnormalities may be profound [16]. These often exhibit symmetrical high voltage slow-wave activity (4–6c/s) throughout the record, extremely large amplitude slow-wave activity (2–3c/s) consistent with our patient [17]. Brain imaging in AS may reveal nonspecific abnormalities such as mild cortical atrophy, loss in white matter volume, or isolated abnormalities in myelinated regions [16,18]. The brain MRI on our case showed microcephaly, corpus callosum dysgenesis, and heterotropia grey matter on the bilateral lateral ventricle. These most likely represent coincidences.

Several genetic tests often used to screen for Angelman syndrome are single nucleotide polymorphism array or methylation-specific multiplex ligation-dependent probe amplification (MS‐MLPA). However, these examinations can only detect maternal deletion, imprinting defects, and paternal UPD but cannot detect point mutations. WES is recently being used to detect pathogenic small nucleotide variants in the UBE3A gene, uniparental isodisomy, and certain large deletions [6]. Initially, we suspected our patient to have epileptic encephalopathy. Therefore, we used WES to find out the underlying etiology of the epileptic encephalopathy and then found the *UBE3A* gene point mutation. WES limitations include its high cost, requiring competent technician to do the testing and the inability to sequence noncoding regulatory or deep intronic regions of known genes that may represent pathogenic variants.

The loss of function of the maternally inherited UBE3A gene causes AS. UBE3A is located on chromosome 15q11–13 and is biallelically expressed throughout the body. However, it is only maternally expressed in the brain. The paternal copy is silenced by the UBE3A-ATS, a long (>600 kb) noncoding RNA antisense transcript [19]. In vitro and in vivo, ASO treatment resulted in a specific reduction of UBE3A-ATS and sustained unsilencing of paternal Ube3a in neurons in mice. In an AS mouse model, partial restoration of UBE3A protein alleviated some cognitive deficits associated with the disease [20].

We report a case with point mutations in the *UBE3A* gene that is only found in 10% of AS. Only a few studies have found the *UBE3A* gene mutation. For example, one study in Brazil reported siblings had frameshift mutations, in Italy found a girl with a splice site mutation. In contrast, a study in Canada found the importance of sequencing in diagnosing pathogenic intronic variants that were not diagnosed using conventional testing [21,22]. The severity of the phenotype is highly dependent on the underlying genetic mechanism. However, our patient had no tremors, jerky motions, or obesity features. This is in accordance with the findings that patients with a point mutation of the *UBE3A* gene have a milder phenotypic, higher developmental, better expressive language skills, and lower seizure prevalence than deletion, UPD, and imprinting defect AS [4,22].

The treatment interventions for our patient were symptomatic. This multi-prong approach is in accordance with another study which stated that the main goals of AS therapy are: 1) to improve gross and fine motor skills, 2) the use of communication therapy such as communication aids (pictures or modified sign language), and 3) administer anticonvulsants for the treatment of any seizures [1].

## 4 Conclusions

This case report is intended to inform clinicians about the clinical features of AS and the importance of WES in diagnosing this rare neurodevelopmental syndrome. WES could detect a rare variant of AS, identified as the point mutation of the UBE3A gene, which cannot be seen with other tests.

## Ethics approval and consent to participate

The Medical and Health Research Ethics Committee of the Faculty of Medicine, Public Health and Nursing, Universitas Gadjah Mada/Dr. Sardjito Hospital ruled the study exempt from approval because this study was a case report (KE/0550/06/2020). The authors attest that full and informed consent was obtained from the patient's parents, who had undergone medical treatment in our hospital.

## Consent for publication

Written and informed consent was obtained from the patient's parents to publish this case report and the associated images.

## Availability of data and material

The datasets used and/or analyzed during the current study are available from the corresponding author on reasonable request.

## Funding

This study was supported by the Ministry of Research and Technology/National Research and Innovation Agency Republic of Indonesia (RISTEK/BRIN) (PDUPT to AT) with contract number 6E1/KP.PTNBH/2021 and 1656/UN1/DITLIT/DIT-LIT/PT/. The funding body had no influence on study design, data analysis, data interpretation, and manuscript writing.

## Authors' contributions

AT, ESH, KI – wrote, designed the study, and edited the manuscript. G-supervised and reviewed the manuscript. APN and MLH – wrote the manuscript, collected and analyzed the clinical data. All the authors read and approved the final manuscript.

## Registration of Research Studies

N/A

## Guarantor

The Guarantor is the one or more people who accept full responsibility for the work and/or the conduct of the study, had access to the data, and controlled the decision to publish

## Consent

We have obtained written and signed consent to issue case reports from patients.

## Declaration of competing interest

The authors declare that they have no competing interest.

## List of abbreviations

ACMG: American College of Medical Genetics and Genomics
AS: Angelman syndrome
DNA: deoxyribonucleic acid
EEG: electroencephalogram
GABA: gamma-aminobutyric acid
MRI: magnetic resonance image
MS-MLPA: methylation-specific multiplex ligation-dependent probe amplification
RNA: ribonucleic acid
UBE3A- ATS: ubiquitin ligase E3A antisense transcript
UPD: uniparental disomy
WES: whole-exome sequencing
