# Supplementary material for: The role of whole exome sequencing in the UBE3A point mutation of Angelman Syndrome: A case report
Source: Ann Med Surg (Lond). 2021 Dec 8;73:103170. doi: 10.1016/j.amsu.2021.103170 (PMC8683671; doi:10.1016/j.amsu.2021.103170)
Supplement: Multimedia component 1 [file mmc1.pdf]

CARE
case report guidelines

CARE Checklist of information to include when writing a case report

(cc) BY-NC-ND

Topic	Item	Checklist item description	Reported on Line
**Title**	1	The diagnosis or intervention of primary focus followed by the words "case report"	1-2
**Key Words**	2	2 to 5 key words that identify diagnoses or interventions in this case report, including "case report"	24-25
**Abstract (no references)**	3a	Introduction: What is unique about this case and what does it add to the scientific literature?	59-61
	3b	Main symptoms and/or important clinical findings	65-66;73-74
	3c	The main diagnoses, therapeutic interventions, and outcomes	98-103
	3d	Conclusion—What is the main "take-away" lesson(s) from this case?	98-103
**Introduction**	4	One or two paragraphs summarizing why this case is unique (**may include references**)	160-164
**Patient Information**	5a	De-identified patient specific information.	N/A
	5b	Primary concerns and symptoms of the patient.	65-66
	5c	Medical, family, and psycho-social history including relevant genetic information	67-68
	5d	Relevant past interventions with outcomes	101-103
**Clinical Findings**	6	Describe significant physical examination (PE) and important clinical findings.	79-85
**Timeline**	7	Historical and current information from this episode of care organized as a timeline	65-67
**Diagnostic Assessment**	8a	Diagnostic testing (such as PE, laboratory testing, imaging, surveys).	89-100
	8b	Diagnostic challenges (such as access to testing, financial, or cultural)	106-109
	8c	Diagnosis (including other diagnoses considered)	98-100
	8d	Prognosis (such as staging in oncology) where applicable	N/A
**Therapeutic Intervention**	9a	Types of therapeutic intervention (such as pharmacologic, surgical, preventive, self-care)	100-103
	9b	Administration of therapeutic intervention (such as dosage, strength, duration)	101-103
	9c	Changes in therapeutic intervention (with rationale)	N/A
**Follow-up and Outcomes**	10a	Clinician and patient-assessed outcomes (if available)	N/A
	10b	Important follow-up diagnostic and other test results	N/A
	10c	Intervention adherence and tolerability (How was this assessed?)	N/A
	10d	Adverse and unanticipated events	N/A
**Discussion**	11a	A scientific discussion of the strengths AND limitations associated with this case report	105-109
	11b	Discussion of the relevant medical literature **with references**.	105-158
	11c	The scientific rationale for any conclusions (including assessment of possible causes)	160-164
	11d	The primary "take-away" lessons of this case report (without references) in a one paragraph conclusion	162-164
**Patient Perspective**	12	The patient should share their perspective in one to two paragraphs on the treatment(s) they received	N/A
**Informed Consent**	13	Did the patient give informed consent? Please provide if requested	**Yes** [v] **No** [ ]
